# Relative and Quantitative Characterization of the Bovine Bacterial Ocular Surface Microbiome in the Context of Suspected Ocular Squamous Cell Carcinoma

**DOI:** 10.3390/ani13121976

**Published:** 2023-06-13

**Authors:** Hannah B. Gafen, Chin-Chi Liu, Nikole E. Ineck, Clare M. Scully, Melanie A. Mironovich, Lauren Guarneri, Christopher M. Taylor, Meng Luo, Marina L. Leis, Erin M. Scott, Renee T. Carter, Andrew C. Lewin

**Affiliations:** 1 Department of Veterinary Clinical Sciences, Louisiana State University, Baton Rouge, LA 70803, USA; hgafen1@lsu.edu (H.B.G.); cliu@lsu.edu (C.-C.L.); nikoleeineck@gmail.com (N.E.I.); cscully@lsu.edu (C.M.S.); mironovich1@lsu.edu (M.A.M.); reneecarter@lsu.edu (R.T.C.); 2 Department of Pathobiological Sciences, Louisiana State University, Baton Rouge, LA 70803, USA; lguarneri1@lsu.edu; 3 Department of Microbiology, Immunology, and Parasitology, School of Medicine, Louisiana State University, New Orleans, LA 70112, USA; ctay15@lsuhsc.edu (C.M.T.); mluo2@lsuhsc.edu (M.L.); 4 Department of Small Animal Clinical Sciences, Western College of Veterinary Medicine, Saskatoon, SK S7N 5B4, Canada; marina.leis@usask.ca; 5 Department of Clinical Sciences, College of Veterinary Medicine, Cornell University, Ithaca, NY 14853, USA; ems462@cornell.edu

**Keywords:** squamous cell carcinoma, microbiome, real-time PCR, sequencing, bovine, discriminant analysis, 16S rRNA gene sequencing

## Abstract

**Simple Summary:**

The ocular surface microbiome is altered in certain disease states. This study aimed to characterize the ocular surface microbiome of cattle with and without ocular squamous cell carcinoma (OSCC), a common eye disease in cattle. From a sample population of ten normal adult female cattle (twenty normal eyes) and nine adult female cattle with unilateral or bilateral OSCC lesions (ten diseased eyes, eight normal eyes), significant quantitative (real-time polymerase chain reaction (RT-PCR)) and relative abundance (16S ribosomal ribonucleic acid (rRNA) gene sequencing) bacterial microbiome alterations were identified based on disease status (normal or OSCC) and geographic location. Quantitative bacterial ocular microbiome discriminant analysis (DA) was capable of accurately categorizing samples based on disease status and geographic location. Quadratic DA, a method based on relatively inexpensive and readily accessible equipment, may be valuable for differentiating between normal and OSCC-affected cattle.

**Abstract:**

The ocular surface microbiome is altered in certain disease states. The aim of this study was to characterize the bovine bacterial ocular surface microbiome (BBOSM) in the context of ocular squamous cell carcinoma (OSCC). The conjunctiva of normal (*n* = 28) and OSCC (*n* = 10) eyes of cows aged 2 to 13 years from two farms in Louisiana and Wyoming were sampled using individual sterile swabs. DNA extraction followed by 16S ribosomal ribonucleic acid (rRNA) gene sequencing and real-time polymerase chain reaction (RT-PCR) were performed to, respectively, assess the relative and absolute BBOSM. Discriminant analysis (DA) was performed using RT-PCR data, and relative abundance analysis was performed using 16S rRNA gene sequencing data. The 11 most abundant phyla in both normal and OSCC-affected cows were identified using 16S rRNA gene sequencing analysis. The relative abundance of Euryarchaeota was found to be significantly lower (*p* = 0.0372) in OSCC eyes compared to normal eyes. Relative abundance differences within and between geographic locations were also identified. Quadratic DA categorized samples as OSCC or normal with 100% sensitivity and 83.3–100% specificity. Relative abundance analysis identified relative BBOSM phylum alterations in OSCC. Quadratic DA can be used to accurately categorize BBOSM from normal and OSCC ocular surface samples.

## 1. Introduction

Ocular squamous cell carcinoma (OSCC), colloquially known as “cancer eye”, is the most common malignant neoplasia affecting the eyes of cattle [1], resulting in significant morbidity, rejection at slaughter, and economic loss [2]. OSCC manifests as irregular tissue proliferation at the region of the corneoscleral junction, nictitating membrane, cornea, and eyelid [3]. OSCC may metastasize to regional lymph nodes and the lungs, though early excision may be curative [4,5]. A microbiome is composed of various microorganisms, such as bacteria, fungi, and viruses, that co-exist simultaneously in the same environment. The normal bacterial microbiome can be altered in the presence of neoplasia in humans [6,7,8,9,10]. Alterations to the normal bacterial microbiome have been identified in the context of squamous cell carcinomas in humans [11,12,13,14]. In humans, dysregulation of the normal resident bacterial ocular surface microbiome is associated with several diseases, including diabetes, dry eye, and allergic conjunctivitis [15,16]. The normal and altered bacterial ocular surface microbiome has been characterized in numerous species, including cats, dogs, pigs, and horses, among others [17,18,19,20,21]. Understanding bacterial microbiome alterations in ocular disease has the potential to inform future diagnostic assays and treatment strategies.

Bacterial relative abundance is frequently assessed using 16S ribosomal ribonucleic acid (rRNA) gene sequencing, which utilizes bacterial conserved and hypervariable regions to classify bacteria within a sample into operational taxonomic units (OTUs) or amplicon sequence variants (ASVs) [22,23,24]. The taxonomic diversity of ASVs within a sample is evaluated for richness and evenness of distribution using different indices, such as Observed (Obs) ASV, Simpson, Shannon, Chao1, Pielou, and Faith’s phylogenetic diversity (PD), to measure a sample’s alpha-diversity [25]. The taxonomic diversity of ASVs between samples (beta-diversity) is evaluated through various pairwise distance metrics such as weighted and unweighted UniFrac analysis [25]. The ocular surface is a relatively low biomass environment, and methods routinely utilized for 16S rRNA gene sequencing relative abundance analysis require various modifications to be suitable for this application [25,26,27].

For absolute characterization of selected elements of the bacterial ocular microbiome at the species level and higher, real-time polymerase chain reaction (RT-PCR) can be utilized [28,29]. In RT-PCR, the number of cycles required to amplify a signal above a specified threshold value (Ct) allows for the estimation of bacterial load. Primers targeting several bacterial families, genera, or species can be simultaneously assessed using RT-PCR [28,29,30]. These bacterial targets can be chosen based on previous clinical information or relative abundance analysis. While primer specificity limits the number of bacterial species detected compared with 16S rRNA gene sequencing, estimation of bacterial quantity provides unique and meaningful insights that otherwise cannot be gained [26,28,29,31].

The bovine bacterial ocular surface microbiome (BBOSM) has been previously investigated in calves [30,32]. In one previous study that utilized 16S rRNA gene sequencing analysis, the relative abundance of *Weeksellaceae*, *Methylobacteriaceae*, and *Mycoplasmataceae* was found to be significantly different on the conjunctival surface of eyes with and without infectious bovine keratoconjunctivitis (IBK) [32]. In addition, clustering of samples based on geographic groupings of animals was observed [32]. A second study used similar methods in an attempt to predict which animals would go on to develop IBK [30]. *Pasteurellaceae*, *Moraxella*, *Mycoplasma*, and *Weeksellaceae* were among the most common families and genera detected in normal cattle in this study [30].

The BBOSM has not yet been studied in the presence of OSCC. This study aimed to assess the BBOSM using both a relative abundance (16S rRNA gene sequencing analysis) and an absolute abundance (RT-PCR) approach. We hypothesized that there would be both relative and absolute abundance differences in the BBOSM in normal and OSCC-affected eyes.

## 2. Materials and Methods

### 2.1. Study Population and Sample Collection

The study was approved by the Louisiana State University Institutional Animal Care and Use Committee (Animal Use Protocol 19-092). Nineteen female cattle (38 eyes) aged 2 to 13 years old from two different geographic locations (Louisiana and Wyoming) were included in the study. Samples were collected between May and October 2021.

A gross ophthalmic examination of all animals was performed by a trained veterinary observer (HBG). Lesions consistent with OSCC [2] and the lesion location (eyelid, nictitating membrane, and limbus) were identified and documented. For all animals, both lower conjunctival fornices were individually sampled by one observer (HBG) with twin swabs (Isohelix DNA Buccal Swab Pack, MidSCi, St. Louis, MO, USA) by vigorously rubbing the conjunctiva for 2–5 s. Swabs were placed in labeled 15 mL sterile centrifuge tubes (VWR, Radnor, PA, USA) prior to being stored on ice. Non-sterile gloves (VWR, Radnor, PA, USA) were worn and changed between each animal during conjunctival swab sample collection. Where possible, samples from normal animals were collected from the same geographic location as those from OSCC-affected animals. Environmental control samples were collected at each geographic location by exposing twin swabs to the air for approximately 5 s with immediate storage in a 15 mL tube on ice. All samples were shipped directly to the LSU School of Veterinary Medicine (LSU SVM) for processing.

### 2.2. Confirmation of OSCC

For confirmation of OSCC in affected animals, an incisional biopsy was collected from the eyelid and nictitating membrane lesions where possible, following swab collection as outlined above. The small size of some lesions precluded safe collection of a confirmatory biopsy; specimens were obtained from 6/10 eyes exhibiting lesions consistent with OSCC. Proparacaine hydrochloride ophthalmic solution (0.5%, Akorn, Inc., Lake Forest, IL, USA) was applied to the ocular surface, followed by povidone-iodine solution (AVRIO Health, Stamford, CT, USA). A regional infiltrative block of 2% lidocaine (VetOne, MWI Animal Health, Boise, ID, USA) was injected prior to the removal of eyelid lesions. An incisional biopsy was performed using a #10 or #15 scalpel blade or scissors. The biopsy specimen was placed in a 10% neutral buffered formalin container for fixation and storage. Biopsy samples fixed in 10% formalin for a minimum of 1 month were embedded in paraffin and cut into 5 µm serial sections, then stained with hematoxylin and eosin for histopathological analysis by a residency-trained veterinary anatomic pathologist (LG).

### 2.3. DNA Extraction

DNA extraction was performed using the DNeasy PowerSoil Pro Kit (QIAGEN GmbH, Hilden, Germany) by following the manufacturer’s instructions. DNA was extracted from each conjunctival swab and from the environmental control samples, as well as from extraction control samples created by replicating the protocol in the absence of swabs. Extractions were performed in a filtered laminar flow cabinet (The Clone Zone, USA/Scientific, Inc., Ocala, FL, USA). The concentrations of each eluted DNA sample were calculated (NanoDrop One Microvolume UV-Vis Spectrophotometer, ThermoFisher Scientific, Waltham, MA, USA) and documented prior to storage at −80 °C.

### 2.4. 16S rRNA Gene Sequencing and Analysis

DNA sequencing was performed by the Louisiana State University School of Medicine Microbial Genomics Resource Group. Two steps of amplification were performed to prepare the sequencing library using the AccuPrime Taq high-fidelity DNA polymerase system (Invitrogen, Carlsbad, CA, USA). Negative controls (DNA extraction and environment) and a positive control (microbial mock community HM-276D, BEI Resources, Manassas, VA, USA) were also processed during amplicon library preparation. The hypervariable V4 region was amplified using 20 ng of genomic DNA and gene-specific primers with Illumina adaptors. The PCR conditions were as follows: 95 °C for 3 min and 25 cycles of 95 °C for 30 s; 55 °C for 30 s and 72 °C for 30 s; and 72 °C for 5 min and holding at 4 °C. PCR products were purified using AMPure XP beads, with beads added at 0.85× the PCR volume. Four µL of purified amplicon DNA from the previous step was amplified for 8 cycles with the same PCR conditions using primers with different molecular barcodes. The indexed amplicon libraries were purified using AMPure XP beads and quantified using Quant-iT PicoGreen (Invitrogen), then normalized and pooled. The pooled library was quantified using the KAPA Library Quantification Kit (Kapa Biosystems, Cape Town, South Africa), diluted, and denatured as per Illumina guidelines. Ten per cent Illumina PhiX was added to the sequencing library as an internal control and to increase the diversity of the 16S rRNA amplicon library. Paired-end sequencing was performed using an Illumina MiSeq (Illumina, San Diego, CA, USA) using the 2 × 250 bp V2 sequencing kit. The sequencing reads were transferred to Illumina’s BaseSpace for quality analysis, and the generated raw FASTQ files were used for further bioinformatics analysis.

Sequencing reads from FASTQ files were imported into R version [33] 4.2.0 and processed with DADA2 [34] version 1.22.0. Read quality profiles were examined to select appropriate trimming and filtering parameters, which were set to truncate reads to 240 bp (both forward and reverse) to remove low-quality tails and trim 20 bp (left) of each read. The standard DADA2 workflow was utilized, including error learning and sample inference for forward and reverse reads, followed by merging of sequence variants. Sequence variants outside of the expected amplicon size of 249 to 256 bp were removed, and chimeric sequence variants were detected and removed using ‘removeBimeraDenovo’. The remaining sequence variants were placed into a sequence table with read counts ranging from 816 to 143,459. Taxonomy was classified using the SILVA database [35], and mapping information was imported to construct a Phyloseq [36] object. Downstream analysis was performed using Phyloseq [36], version 1.38.0. The suspected contaminant ASV was identified and removed using Decontam [37] version 1.14.0 with the prevalence method. An abundance filter was used to filter out the remaining ASVs with a mean of less than 10^−4^ across all samples.

### 2.5. RT-PCR Processing and Analysis

RT-PCR for selected bacterial families and species (Table 1) was performed using remaining eluted DNA and PerfeCTa^®^ SYBR^®^ Green FastMix^®^, ROX™ (VWR, Radnor, PA, USA). Primer pairs for bacterial targets were chosen based on previous clinical reports and exploratory 16S rRNA gene sequencing relative abundance analyses obtained from cattle with eye disease [30,32,38]. Eluted DNA was diluted with molecular grade water (VWR, Radnor, PA, USA) to concentrations standardized between the eyes of the same animal, with an overall range of 0.63 to 16.07 ng/µL. Each DNA sample was individually combined in triplicate with nine different primer pairs. The RT-PCR panel performed on each sample included the following primer (IDT, Coralville, IA, USA) targets: bovine GAPDH [39], *Moraxella bovis* [38], *Moraxella bovoculi* [38], *Mycoplasma* [40], *Pasteurellaceae* [41], *Prevotellaceae* [42], *Staphylococcus* [43], *Weeksellaceae*, and ‘universal bacteria’ [29]. The bovine GAPDH primer was utilized as a reference for host DNA in proportion to the total bacterial DNA extracted, while the universal bacteria primer was used for data normalization. Molecular grade water (VWR, Radnor, PA, USA) and an *Escherichia coli* standard (10 ng/µL, Sigma-Aldrich, Saint Louis, MO, USA) were used as negative and positive controls, respectively. The *E. coli* standard was plated in triplicate with both ‘universal bacteria’ primers and *E. coli* primers [44] to allow for standardization between runs and relative quantification of non-*E. coli* bacterial groups. The RT-PCR run included a hold stage of 50 °C for 2 min and 95 °C for 10 min; a PCR stage of 40 cycles of 95 °C for 15 s followed by 60 °C for 10 min; a continuous melt curve stage of 95 °C for 15 s and 60 °C for 15 s; and a dissociation step of 95 °C for 15 s. The RT-PCR data was expressed as the log amount of DNA (ag) for each primer pair per 10 ng of isolate total DNA using an E. coli standard on the same RT-PCR plate. The Ct values of each primer set were normalized by universal bacteria Ct values within each sample for further analyses.

### 2.6. Statistical Analysis

All statistical analyses were performed using commercial software (JMP Pro [45] and R Statistical Software [33] v4.1.3). Associations between eye samples (right or left), geographic location, age, and OSCC status were checked via the chi-squared test. Abundance, alpha diversity from 16S rRNA gene sequencing, and log DNA concentration from RT-PCR were analyzed with a t test or a Mann–Whitney test against disease status (normal or OSCC) or location. For beta diversity, standard weighted UniFrac analysis, unweighted UniFrac analysis, and Bray–Curtis analysis were evaluated via permutational multivariate analysis of variance (PERMANOVA) using vegan R package 2.6.2 [46]. Quadratic discriminant analyses (QDA) were used to categorize combined RT-PCR data (normalized Ct values for 8 primer sets) by disease status. Linear discriminant analysis (LDA) was used to categorize combined RT-PCR data by geographic location (Louisiana or Wyoming). Two training sets of 30 random samples were used to generate the QDA model. A validation set of 8 samples was randomly generated via the sample function in R for normal and OSCC samples. For both the QDA and LDA models, primer pairs were removed individually to evaluate the relative contribution of each primer to the model. Statistical significance was at *p* < 0.05.

## 3. Results

### 3.1. Sampling Demographics

A total of 38 eyes (28 normal, 10 OSCC) from 19 cows (all adult females; ten unaffected, nine with unilateral or bilateral OSCC lesions) were included in this study. The median age of the cows sampled was 6 years (range 2–13 years). Nine animals were between the ages of 1 year and 5 years, while 10 cows were 6 years or older. Increasing age (6 years or older) was significantly associated with a higher likelihood of OSCC disease status (*p* = 0.0376). Samples were taken from one cow with unilateral OSCC lesions in Louisiana; all other samples were taken from cattle at a single farm in Wyoming. One environmental negative control sample was obtained at each geographic location.

Eighteen Hereford cattle and one Hereford crossed with Red Angus were sampled. Nine cattle had lesions consistent with OSCC: eight cows had unilateral ocular lesions, while one cow had bilateral ocular lesions consistent with OSCC. Two OSCC lesions were located on the nictitating membrane only; 3/10 OSCC lesions were associated with the corneoscleral junction of the globe; 3/10 OSCC lesions were associated with the eyelids; and 2/10 eyes had lesions on both the upper eyelid and nictitating membrane. Histopathology of 6/6 lesions biopsied confirmed OSCC. Histologic alterations characteristic of OSCC were noted in all biopsy samples, including neoplastic polygonal squamous epithelial cells arranged in anastomosing trabeculae and islands with moderate to marked anisocytosis and anisokaryosis, as well as variable dyskeratosis and the formation of keratin pearls. 

### 3.2. 16S rRNA Gene Sequencing

#### 3.2.1. Bacterial Population Composition

**Figure 1:**
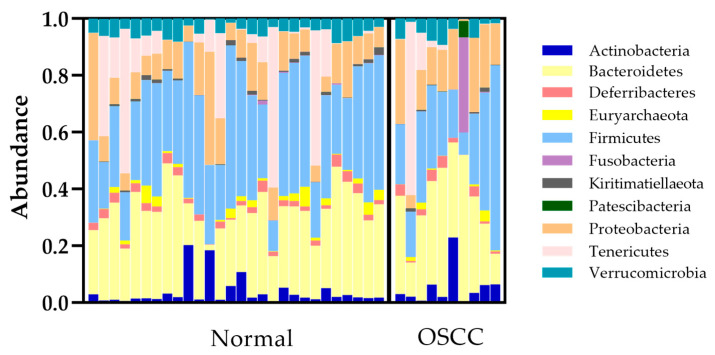
Bacterial microbiome composition of normal and OSCC-affected eyes at the phylum level. Eleven different phyla encompassing 120 ASVs were detected on the conjunctival surface of normal and OSCC-affected eyes. OSCC = ocular squamous cell carcinoma.

From 41 total samples (38 conjunctival swabs, two environmental swabs, and one sequencing control), 9768 ASVs were sequenced. With potential contaminants (ASVs encompassing less than 0.001% frequency) removed, 1079 ASVs were sequenced. An even sequencing depth of 120 ASVs for the 38 conjunctival swab samples was then graphed (Figure 1). The 11 most abundant phyla, encompassing 120 ASVs, included Actinobacteria, Bacteroidetes, Deferribacteres, Euryarchaeota, Firmicutes, Fusobacteria, Kiritmatiellaeota, Patescibacteria, Proteobacteria, Tenericutes, and Verrucomicrobia. The three most abundant phyla recorded within both the control and OSCC populations included Bacteroidetes, Firmicutes, and Proteobacteria (Table 2). Among the 11 most abundant phyla, only Euryarchaeota was found to have a significantly lower relative abundance in OSCC eyes compared to normal eyes (*p* = 0.0372). The relative abundance of Euryarchaeota, Kiritimatiellaeota, and Proteobacteria was significantly different between the two geographic locations (*p* = 0.0057). (Table 2).

#### 3.2.2. Alpha Diversity Analysis

**Figure 2:**
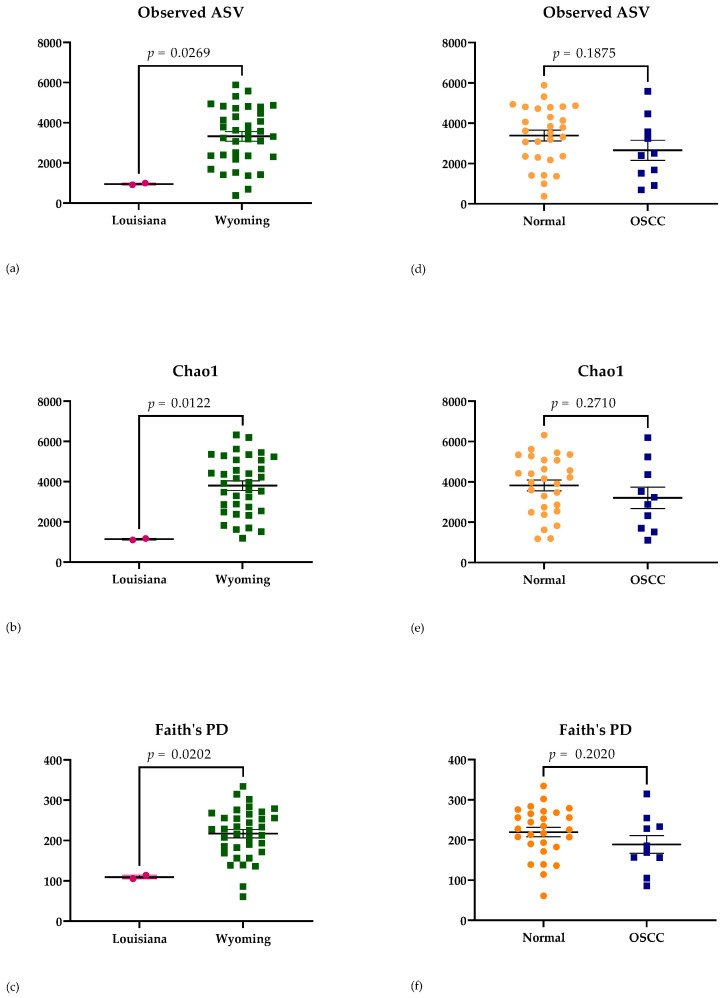
Alpha diversity of sequenced samples using Obs ASV, Chao1, and Faith’s PD. Significant differences in alpha diversity indices were observed with respect to geographic location, as shown in (**a**–**c**). No significant differences in the same alpha diversity indices were observed with respect to disease status, as shown in (**d**–**f**). OSCC = ocular squamous cell carcinoma.

Indices chosen to evaluate the alpha diversity of samples included Obs ASV (richness of microbial communities present [21]), Chao1 (richness at full sequence depth [21]), and Faith’s PD (the sum of the branch lengths of the phylogenetic tree connecting each species [47]). Significance differences in alpha diversity were detected based on geographic location using Obs ASV (*p* = 0.0239), Chao1 (*p* = 0.0122), and Faith’s PD (*p* = 0.0202) (Figure 2). A significant difference in sample alpha diversity was not attributed, however, to disease status (Obs ASV *p* = 0.1875; Chao1 *p* = 0.2710; and Faith’s PD *p* = 0.2020) (Figure 2). No significant difference in sample diversity was attributed to left vs. right eye (Obs ASV *p* = 0.6126; Chao1 *p* = 0.6467; and Faith’s PD *p* = 0.6533), age (Obs ASV *p* = 0.6544; Chao1 *p* = 0.7421; and Faith’s PD *p* = 0.5867), or breed (Obs ASV *p* = 0.9576; Chao1 *p* = 0.8390; and Faith’s PD *p* = 0.9941).

#### 3.2.3. Beta Diversity Analysis

**Figure 3:**
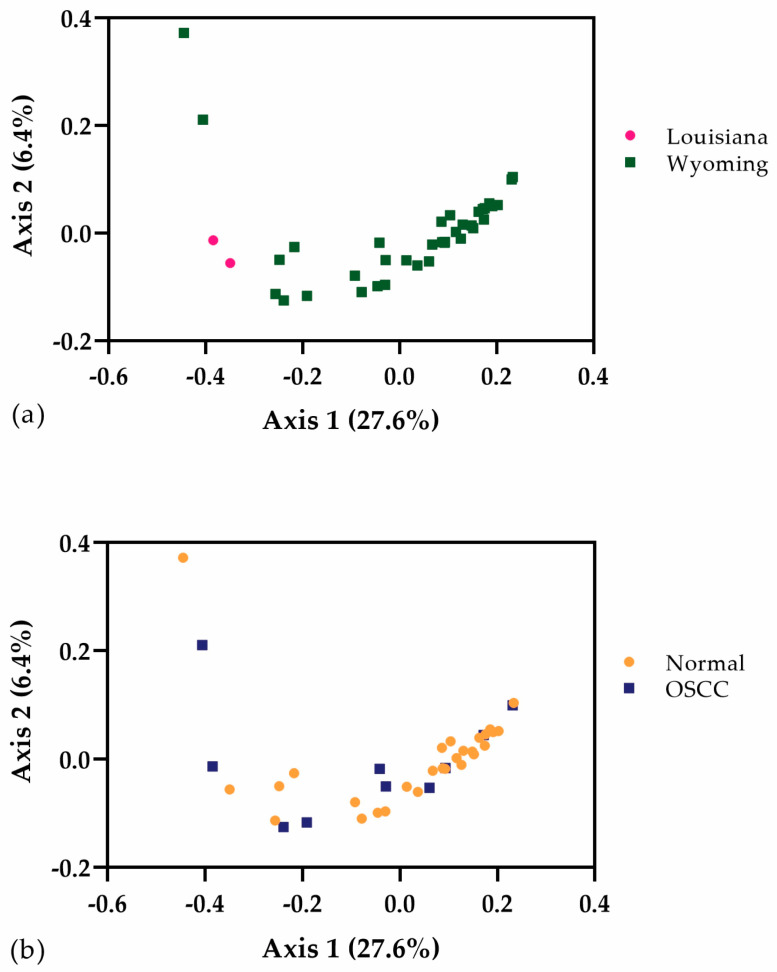
Unweighted UniFrac principal coordinates analysis for geographic location (**a**) and disease status (**b**). Significance was found between geographic locations (*p* = 0.006) (**a**) but not by disease status (*p* = 0.211) (**b**).

Beta diversity was evaluated using standard weighted UniFrac analysis, unweighted UniFrac analysis, and Bray–Curtis analysis. Comparisons were made between left vs. right eyes, geographic location (Louisiana or Wyoming), age (1 to 5 years or over 6 years), breed (Hereford or Hereford cross), and disease status (normal or OSCC). Significant differences between geographic locations (*p* = 0.006) (Figure 3) were found only using unweighted UniFrac analysis. Significant differences in beta diversity were not attributed to other variables, including disease status (*p* = 0.211) (Figure 3).

### 3.3. RT-PCR Analysis

#### 3.3.1. RT-PCR Results

A total of 38 DNA samples were prepared and processed using RT-PCR, with the absolute DNA quantities shown in Table 3. The quantity of *Pasteurellaceae* was significantly higher in OSCC-affected eyes compared to normal eyes (*p* = 0.0279). When comparing geographic locations, *Moraxella bovis*, *Mycoplasma*, and *Prevotellaceae* were found in significantly lower quantities in samples from Louisiana compared to Wyoming (*p* = 0.0341, 0.0057, and 0.0014, respectively) (Table 3).

#### 3.3.2. Quadratic and Linear Discriminant Analyses (QDA/LDA)

The Ct values of eight primer sets (Table 1) were normalized by the universal bacterial primers for the same sample. The data was further analyzed by discriminant analysis for disease status (normal or OSCC) and geographic location (Louisiana or Wyoming). Due to the unbalanced sample size at each location, only 30 samples were used to construct the canonical variable table. Two randomized validation sets (*n* = 8) were used for disease status. The standardized canonical coefficients from two different training sets for disease status and one for geographic location are listed in Table 4.

Quadratic discriminant analyses (QDA) were used to separate disease status (OSCC or normal) using a training set composed of 30 samples (Table 4). The QDA generated from the training set performed well on the validation sets, resulting in 100% sensitivity and 83.3–100% specificity. *Pasteurellaceae* was primarily associated with the canonical variable for disease status QDA, followed by *Staphylococcus* (Table 4). Removal of any one of the eight primer sets resulted in significantly reduced QDA model performance on the validation sets (Table S1).

Linear discriminant analysis (LDA) was used to separate samples by geographic location using 38 samples. Only one sample from Wyoming was misclassified by the LDA (2.6%; Table 4). *Mycoplasma* was primarily associated with the canonical variable for geographic location LDA, followed by *Staphylococcus* and *Prevotellaceae*. Only the removal of the *Mycoplasma* primer set resulted in significantly reduced LDA model performance (Table S2).

## 4. Discussion

The results presented herein provide evidence of BBOSM alterations in the context of OSCC. These results support existing evidence that tissue microbiomes are altered in the context of neoplasia [6,7,8,9,10,11]. Ocular disease in cattle is infrequently studied despite a population of 98.8 million cattle heads in the United States [48]. Bovine ocular disease causes both significant morbidity and economic loss for producers [2]. Diagnosing and treating cattle with OSCC is challenging, as many of these animals graze on open pasture throughout the year with limited daily observation. The benefits of an earlier diagnosis are likely to include improved treatment outcomes and reduced economic loss.

In this study, 16S rRNA gene sequencing and relative abundance analysis identified a single phylum (Euryarchaeota) at a lower abundance in OSCC-affected eyes. Euryarchaeota is a methanogenic archaea found within the guts of over 175 animal species [49] and humans [50]. Previously thought to be pathogenic, these microorganisms are now thought to play a role in homeostasis [50], though their function in livestock species remains mostly unknown [51]. Further studies are necessary to explore the relationship between Euryarchaeota and bovine OSCC. While geographic differences in the relative abundance of Kiritimatiellaeota, Proteobacteria, and Euryarchaeota were also identified, these findings are extremely likely to be primarily driven by an uneven sample size.

A recent study described significant bacterial abundance differences in the oral microbiome of saliva from human patients with and without oral squamous cell carcinoma [11]. The study noted a marked decrease in the abundance of Firmicutes and a marked increase in the abundance of Fusobacteria and Bacteroidetes in the presence of oral squamous cell carcinoma [11]. We detected a non-significant trend for decreased Firmicutes in OSCC samples (Table 2), consistent with this previous study. In addition, though not significant, we also detected a trend for elevations in Bacteroidetes in OSCC samples compared to normal samples (Table 2), similar to the cited study. A trend for elevations in Fusobacteria in OSCC samples was not detected in the present study.

While useful as an exploratory technique, 16S rRNA gene sequencing analysis was found to be mostly unsuitable for the categorization of samples by disease status in our study population. For example, we found that three separate relative abundance beta diversity indices demonstrated no difference between normal eyes and OSCC-affected eyes. While 16S rRNA gene sequencing analysis has been widely utilized for the investigation of altered microbiome composition in high biomass environments such as the gut, these techniques are less widely used in low biomass environments such as the ocular surface [27]. Recognizing this, we deliberately utilized methods to ensure that the potentially significant impact of contamination was accounted for and prevented, where possible. Negative controls were acquired from the sampling environments and DNA extraction kit. Positive and negative controls were utilized during 16S rRNA gene sequencing, with subsequent subtractive analysis performed. All laboratory-based sample handling was performed in a laminar flow hood to prevent contamination. Despite these precautions, some degree of sample contamination is inevitable.

Recent methods of bacterial microbiome quantification using RT-PCR to create a gastrointestinal dysbiosis index (DI) have been described and validated [28,29,31,52,53]. This approach can provide clinically useful insights by accounting for unavoidable variation in the total amount of bacterial DNA recovered from the target tissue or material and providing quantification of bacterial genera, families, and/or species of interest [52,53]. In addition, specific family and species resolution can be achieved using RT-PCR to identify and quantify bacterial targets. Indeed, our results demonstrate the utility of such an approach, as the QDA model was able to readily categorize samples based on disease status. The combination of bacterial targets appears to be critical to the success of the QDA model, as the removal of individual components markedly reduced sensitivity and specificity. 

Given evidence from previous studies demonstrating alterations to the associated bacterial microbiome in the context of neoplasia [6,7,8,9,10,11,12,13,14], as well as evidence from studies demonstrating changes to the human bacterial ocular surface microbiome in certain disease states [15,16], the translational potential of the present study should not be overlooked. In human patients, ocular surface squamous neoplasia yields invasive squamous cell carcinoma at its end stage, resulting in vision loss and death in severe cases [54,55,56]. Further research characterizing the bacterial ocular surface microbiome in the context of ocular squamous cell carcinoma in humans may be pursued in a similar manner to the present study to inform new diagnostic assays and treatment options.

Limitations of this study include a relatively small sample size and an uneven geographic distribution of samples. Geographic significance in relative and quantitative BBOSM alterations was not unexpected due to the small sample size at one location (Louisiana: *n* = one cow, two eyes) compared to the second location (Wyoming: *n* = 18 cows, 36 eyes). In addition, 4/10 eyes diagnosed with OSCC had lesions that were too small to be safely biopsied. Therefore, the OSCC diagnosis could not be confirmed by histopathology in these four eyes. However, clinical diagnoses for these eyes were made by a trained veterinarian and confirmed by a board-certified veterinary ophthalmologist.

## 5. Conclusions

The results presented herein provide evidence of BBOSM alterations in the context of OSCC. Relative abundance-based analysis identified 11 core BBOSM phyla. One of these phyla (Euryarchaeota) was found in significantly lower abundance in OSCC-affected eyes. RT-PCR and subsequent QDA allowed for the categorization of disease status with a high degree of sensitivity and specificity.

## Figures and Tables

**Table 1:** Primers used for the RT-PCR panel. Please note that the ‘universal bacteria’ [29] and the *Escherichia coli* [44] primer sets were used for normalization purposes, and these values were not included in the discriminant analysis models.

Target	Forward Primer	Reverse Primer	Reference
Bovine GAPDH	CCTGGAGAAACCTGCCAAGT	GCCAAATTCATTGTCGTACCA	[39]
*Moraxella bovis*	GGTGACGACCGCTTGTTT	ATCATCGCCTTCATCTCCAG	[38]
*Moraxella bovoculi*	GGTGATATTTATCATGAAGTTGTGAAA	TYTCAATTCATAATCACGATACTCAAG	[38]
*Mycoplasma*	TGCACCATCTGTCACTCTGTTAACCTC	ACTCCTACGGGAGGCAGCAGTA	[40]
*Staphylococcus*	GGCCGTGTTGAACGTGGTCAAATCA	TIACCATTTCAGTACCTTCTGGTAA	[43]
*Pasteurellaceae*	CATAAGATGAGCCCAAG	GTCAGTACATTCCCAAGG	[41]
*Prevotellaceae*	GGTTCTGAGAGGAAGGTCCCC	TCCTGCACGCTACTTGGCTG	[42]
*Weeksellaceae*	ATCCAGCCATCCCGCGT	CTGCTGGCACGGAGTTAGC	None; novel
Universal bacteria	CCTACGGGAGGCAGCAGT	ATTACCGCGGCTGCTGG	[29]
*Escherichia coli*	CCGATACGCTGCCAATCAGT	ACGCAGACCGTAGGCCAGAT	[44]

**Table 2:** Summary of 16S rRNA gene sequencing results at the phylum level. An asterisk (*) denotes significant differences in % abundance by disease status or location. OSCC = ocular squamous cell carcinoma.

	Median (Min–Max) Abundance Composition (%)			
Phylum	Disease Status		Location	
	Normal (*n* = 28)	OSCC (*n* = 10)	Louisiana (*n* = 2)	Wyoming (*n* = 36)
Actinobacteria	1.75 (0.49–20.17)	3.19 (0.25–22.85)	2.92 (2.86–2.98)	1.90 (0.25–22.85)
Bacteroidetes	29.27 (1.88–45.95)	33.69 (10.70–51.72)	28.62 (22.61–34.62)	30.23 (1.88–51.72)
Deferribacteres	2.08 (0.16–4.29)	1.87 (0.06–4.23)	3.25 (2.55–3.95)	2.10 (0.06–4.29)
Euryarchaeota	1.26 (0.02–6.90) *	0.40 (0.00–3.81) *	0.01 (0.00–0.02) *	1.15 (0.01–6.90) *
Firmicutes	32.88 (10.76–57.51)	23.69 (7.89–65.20)	25.21 (21.27–29.15)	29.35 (7.89–65.20)
Fusobacteria	0.04 (0.00–1.46)	0.01 (0.00–33.53)	0.00 (0.00–0.00)	0.03 (0.00–33.53)
Kiritimatiellaeota	0.60 (0.00–2.61)	0.23 (0.00–1.51)	0.00 (0.00–0.00) *	0.52 (0.00–2.61) *
Patescibacteria	0.00 (0.00–0.06)	0.00 (0.00–5.70)	0.00 (0.00–0.00)	0.00 (0.00–5.70)
Proteobacteria	9.56 (5.39–39.85)	14.58 (0.59–29.80)	33.75 (29.80–37.71) *	10.07 (0.59–39.85) *
Tenericutes	1.97 (0.06–56.49)	0.20 (0.02–60.99)	0.16 (0.16–0.17)	1.36 (0.02–60.99)
Verrucomicrobia	4.85 (0.38–8.59)	4.38 (0.15–9.26)	6.08 (4.94–7.21)	4.62 (0.15–9.26)

**Table 3:** Summary of absolute DNA quantities. The values shown are the log amount of DNA (ag) per 10 ng of isolated total DNA. An asterisk (*) denotes significant differences in % abundance by disease status or location. OSCC = ocular squamous cell carcinoma.

	Median (Min–Max) Log DNA (ag) per 10 ng Isolated DNA			
Target Primer	Disease Status		Location	
	Normal (*n* = 28)	OSCC (*n* = 10)	Louisiana (*n* = 2)	Wyoming (*n* = 36)
Bovine GAPDH	7.39 (7.11–7.59)	7.46 (6.96–7.57)	7.44 (7.42–7.47)	7.39 (6.96–7.59)
*Moraxella bovis*	3.45 (0.00–4.87)	3.25 (2.42–4.26)	2.71 (2.42–3.00) *	3.38 (0.00–4.87) *
*Moraxella bovoculi*	3.11 (0.00–5.75)	3.50 (0.00–6.43)	3.83 (2.27–5.38)	3.11 (0.00–6.43)
*Mycoplasma*	6.07 (3.54–8.06)	5.08 (0,00–8.42)	1.79 (0.00–3.58) *	5.98 (3.46–8.42) *
*Staphylococcus*	4.13 (0.00–7.74)	5.02 (0.00–7.46)	5.62 (5.24–6.00)	4.19 (0.00–7.74)
*Pasteurellaceae*	4.27 (3.72–7.22) *	5.63 (3.69–7.10) *	5.09 (4.51–5.66)	4.34 (3.69–7.22)
*Prevotellaceae*	7.00 (5.55–7.85)	8.82 (4.97–8.98)	5.26 (4.97–5.55) *	7.00 (5.85–8.99) *
*Weeksellaceae*	6.09 (5.09–7.72)	6.22 (5.19–7.10)	5.56 (5.19–5.93)	6.20 (5.09–7.72)

**Table 4:** Standardized canonical coefficients and performance of quadratic and linear discriminant analysis. ^a^ = values from the training set. ^b^ = values from the validation set.

	Disease Status (*n* = 30)		Location (*n* = 38)
	Training Set #1	Training Set #2	
**Canonical Standardized Coefficients**			
Bovine GAPDH	0.1824	0.3792	0.3741
*Moraxella bovis*	−0.4893	0.1722	0.2181
*Moraxella bovoculi*	0.1398	0.2992	0.2206
*Mycoplasma*	0.4204	0.2334	−0.7565
*Staphylococcus*	0.5929	−0.3859	−0.6343
*Pasteurellaceae*	−0.9643	0.9771	0.1322
*Prevotellaceae*	0.0860	0.3149	−0.5426
*Weeksellaceae*	0.3759	0.7158	0.2818
**Sensitivity** (**%**)	100 ^a^, 100 ^b^	100 ^a^, 100 ^b^	
**Specificity** (**%**)	95.5 ^a^, 83.3 ^b^	100 ^a^, 100 ^b^	
**Misclassified** (**%**)			2.6

## Data Availability

The data presented in this study are openly available in the National Center for Biotechnology Information Sequence Read Archive, reference number BioProject ID PRJNA932729.

## Supplementary Materials

The following supporting information can be downloaded at: https://www.mdpi.com/article/10.3390/ani13121976/s1, Table S1: QDA Training Sets and Validation Sets, and Table S2: LDA Misclassification.
